# Evidence-Based Management and Controversies in Blunt Splenic Trauma

**DOI:** 10.1007/s40719-017-0074-2

**Published:** 2017-02-09

**Authors:** D. C. Olthof, C. H. van der Vlies, J. C. Goslings

**Affiliations:** 1 Trauma Unit, Academic Medical Center, Meibergdreef 9, 1105 AZ Amsterdam, Netherlands; 2 Division of Trauma Surgery, Maasstad Hospital, Maasstadweg 21, 3079 DZ Rotterdam, Netherlands

**Keywords:** Blunt abdominal trauma, Splenic injury, Non-operative management, Embolisation, Splenic function

## Abstract

**Purpose of review:**

The study aims to describe the evidence-based management and controversies in blunt splenic trauma.

**Recent findings:**

A shift from operative management to non-operative management (NOM) has occurred over the past decades where NOM has now become the standard of care in haemodynamically stable patients with blunt splenic injury. Splenic artery embolisation (SAE) is generally believed to increase the success rate of NOM. Not all the available evidence is that optimistic about SAE however. A morbidity specifically related to SAE of up to 47% has been reported. Although high-grade splenic injury is a prognostic factor for failure of NOM, an American research group has published a study in which NOM is performed in over half of haemodynamically stable patients with grade IV or V splenic injury without leading to an increased morbidity (in terms of complications) or mortality. Another area of current investigation in the literature is the exact indication for SAE. Although the generally accepted indication is the presence of vascular injury, a topic of current investigation is whether there might be a role for pre-emptive embolisation in patients with high-grade splenic injury. On the other hand, evidence is also emerging that not all blushes require an intervention (small blushes <1 or 1.5 cm do not). Lastly, the available evidence shows that splenic function is preserved after embolisation, and therefore, the routine administration of vaccinations seems not to be necessary. There might be a difference between proximal and distal embolisations; however, with regard to splenic function, in favour of distal embolisation.

**Summary:**

Nowadays, NOM is the standard of care in haemodynamically stable patients with blunt splenic injury. The available evidence (although with a relatively small number of patients) shows that splenic function is preserved after NOM, a major advantage compared to splenectomy. SAE is used as an adjunct to observation in order to increase the success rate of NOM. Operative management should be applied in case of haemodynamic instability or if associated intra-abdominal injuries requiring surgical treatment are present. Patient selection (which patient can be safely treated non-operatively, does every blush needs to be embolised?, which patients might be better off with direct operative intervention given the patient and injury characteristics) is an ongoing subject of further research. Future studies should also focus on long-term outcomes of patients treated with embolisation (e.g. total number of lifetime infectious episodes requiring antibiotic treatment or hospital admission, quality of life).

## Introduction

Nearly 199,800 people die from injury each year—1 person every 3 min [1]. Trauma is the most common cause of death in people under the age of 45 years and in the top 3 leading causes of death in all age groups [2]. The prevalence of intra-abdominal injury amongst patients presenting to the emergency department is around 15%, and the spleen is the most commonly injured organ in blunt abdominal trauma [3].

In the past decades, the treatment of patients with blunt splenic injury has shifted from operative to non-operative management (NOM). Operative management was applied because of the belief that the spleen was an organ without function, that it could not heal on its own and could rupture in a later stage. Also, the mortality rate of patients who were not operated was unacceptably high, whereas patients who were operated had a change of survival [4]. Nowadays, splenic artery embolisation (SAE) has become available and well studied. This has led to NOM being the treatment of choice. NOM has several advantages over operative management. First of all, the risk of overwhelming post-splenectomy infection, an infection caused by encapsulated bacteria, which occurs after only 0.5% of all splenectomies in trauma patients but carries a mortality rate of around 50 to 70% [5], is avoided. Second, in case of failure of NOM, there is the possibility of a second non-operative re-intervention, for example an attempt for SAE after failure of observation or proximal embolisation after failure of distal embolisation. Third of all, possible surgery-related complications are avoided. Lastly, a shorter hospitalisation period and a concomitant reduction in costs have been reported in the literature [6]. However, disadvantages of NOM exist as well. There is a risk of delayed splenic rupture, the possibility of re-bleeding and minor or major complications of embolisation might occur (e.g. splenic infarction, splenic abscess, contrast-induced renal insufficiency, coil migration or pleural effusion [7, 8]), and the fact that no (intra-operative) view can be obtained of other abdominal organs is an important disadvantage.

In this review, evidence-based management and controversies in blunt splenic trauma are discussed and future perspectives are highlighted.

## Non-operative Management

NOM consists of close observation of the patient and can be supplemented with SAE if necessary. Observational management involves admission to a medium or intensive care unit with close monitoring of vital signs, bed rest, frequent monitoring of hemoglobin concentration, and serial abdominal examinations [9]. Therefore, the first important necessities in order to safely perform NOM are facilities with continuous monitoring of vital signs, the possibility for transfusion, and the manpower for re-assessment of the patient (several times a day). Also, the hospital should have unlimited access to a CT scanner with intravenous contrast administration, preferably on or close to the trauma room/emergency department [10•]. Furthermore, interventional radiological facilities, i.e. 24-h coverage of an experienced interventional radiologist, and all the required resources should be available. If a hospital is not able to meet these requirements, NOM should not be chosen over surgical management.

## Splenic Artery Embolisation

Indications for SAE are the presence of vascular injury such as contrast extravasation (i.e. a blush), a cut-off, pseudoaneurysms, or arteriovenous fistula. Different types of embolisation exist: proximal (arteria lienalis) or distal (also called selective) embolisation.

Embolisation is generally believed to increase the success rate (outcome: splenic salvage rate) of observation [11]. However, the true additional value of SAE has not been well defined yet. According to Harbrecht et al. [12], improvement in the success rate of NOM of patients with blunt splenic injuries over time is caused, in part, by an increase in the detection of relatively minor splenic injuries by qualitatively better CT scanners. The results of the propensity score analysis [13] suggest that the improvement in success might be attributed to the use of SAE, although there was no significant difference between SAE and observation alone with regard to successful treatment after correction for confounders.

More recently, critical evidence appeared with regard to SAE. Chastang et al. [14] questioned the indication for SAE because of the high overall morbidity of 44% (the authors investigated both morbidity related to splenic injury and severity, morbidity related to associated trauma, and morbidity specifically related to treatment). In their study, the morbidity specifically related to SAE was 47% (7 out of 15 patients treated with embolisation) and consisted of major splenic infarctions, probably leading to functional asplenism and post-embolisation syndromes (hyperthermia, hyperalgia without splenic infarction). Although the results are based on small numbers, a description of the years of experience of the interventional radiologists is lacking, and this high number of complications has not been described by other authors they are alarming and should be put into perspective in future comparative studies. Furthermore, the most important early disadvantage of SAE is not the above-described complications but the possibility of re-bleeding (i.e. failure of NOM).

## Failure of NOM

The failure rate of NOM is around 10%. Early identification of patients at high risk for failure of NOM (eventually requiring a re-intervention, (repeat) SAE, or delayed splenectomy) is essential since delay in recognition and treatment of late splenic ruptures leads to increased resource use, morbidity, and mortality (preventable deaths) [15, 16]. Many prognostic factors for failure have been investigated in the literature, such as grade of splenic injury, the presence of a (large) haemoperitoneum, contrast extravasation at time of admission, high Injury Severity Score (ISS), lower admission systolic blood pressure (SBP), transfusion of >1 packed cell, and the presence of traumatic brain injury. In a study with almost 15,000 patients, Peitzman et al. reported that the failure rate of NOM increased with grade of injury from 4.8% for patients with American Association for the Surgery of Trauma (AAST, see Table 1 in the Appendix) grade I injury, 9.5% for grade II, 19.6% for grade III, 33.3% for grade IV, and 75.0% for grade V, respectively. Higher failure rates with increasing grade of splenic injury were also found by Chastang et al. [14] in a prospective, multicentre study. In a systematic review, strong evidence was found that splenic injury grade ≥3, ISS ≥25, and age ≥40 years affect the outcome of NOM [17]. Awareness is needed because in the presence of these factors, NOM is more likely to fail. Limited evidence was available for a lowered GCS score/the presence of traumatic brain injury.

Although NOM is more likely to fail in patients with high-grade splenic injury, high-grade splenic injury (in haemodynamically stable patients) is not necessarily a contraindication for NOM. Scarborough et al. recently published data of 1516 patients (propensity-matched cohort) with high-grade splenic injury (grades IV and V) in which NOM was compared to immediate splenectomy. NOM was attempted in over half of the patients (52.2%). Interestingly, in approximately 20% of the patients with a NOM attempt, splenectomy was ultimately required. While increasing patient age, hypotension on admission, the presence of any underlying bleeding disorder, and grade V splenic injury (compared to grade IV) increased failure rate, the only protective factor against failure of NOM was the use of SAE. Delay in operative management (in other words, failure of NOM) did not affect mortality (6.4% in failure of NOM group vs 16.4% in immediate splenectomy group) or the number of (non)infectious complications, but the hospital length of stay was significantly longer compared to patients undergoing immediate splenectomy [18•].

An eventual further extension of SAE (not only in case of the presence of vascular injury) is currently being investigated by Gaarder et al. from Oslo. They are preparing a randomised controlled (multicentre) trial in which failure rate (due to splenic bleeding) of NOM is compared between haemodynamically stable patients with high-grade (IV or V) splenic injury undergoing pre-emptive SAE and patients not undergoing SAE.

As important as recognising the risk of failure is being aware of the time interval between trauma and failure. Different research groups have investigated this topic. Peitzman et al. [19] reported that over 85% of the failures occurred in the first 72 h after injury (data of almost 15,000 patients). Smith et al. analysed over 23,000 patients and found that over 95% of failures occurred within the first 3 days and that 2 additional days of hospital admission only captured 1.5% more failures [20••]. Zarzaur et al. performed a prospective study in which the risk of splenectomy (after a minimum of 24-h observation and non-operative attempt) was 0.27% in a time period of 180 days (total follow-up duration) with a median hospital length of stay of 6 days (range 3 to 11). They state that for grade II to V splenic injury, close observation (in hospital or as an outpatient with good instructions) is needed for a total of 10–14 days [21]. Awareness with regard to time to failure is important because it influences the duration of hospital admission. In the Delphi study (expert consensus study), agreement was reached that optimal duration of hospital admission consists of 1–3 days of admission to a monitored setting followed by 1–3 days of admission to a surgical ward [22].

The value of routine follow-up imaging has been debated in the literature. Some authors use routine follow-up CT scanning (at 48 h) to decrease the failure rate of NOM [14, 23] or in order to confirm healing [24] whilst others do only support it routinely [22] or not at all [25]. Serial CTs may be considered for high-level athletes on a case-by-case basis [26].

## Splenic function

The spleen has both an haematological and immunological function. With regard to its immunological function, it is involved in the antibody response against infection, most importantly against encapsulated bacteria such as *Streptococcus pneumoniae*, *Haemophilus influenzae* type B, and *Neisseria meningitides* group C [27, 28]. One of the biggest advantages of NOM is preservation of splenic function. Different studies have investigated splenic function after SAE, most of them concluding that immunocompetence of the spleen is preserved [29–32]. However, no gold standard exists for measuring splenic function and the majority of the studies performed are retrospective in nature and/or included a relatively small number of patients. Olthof et al. performed a prospective study in which splenic function of embolised patients was compared to function of both healthy controls and patients who underwent splenectomy. Splenic function was assessed (amongst others) by the antibody response to pneumococcal 23-valent polysaccharide vaccine (the ratio of the IgG antibody level post vaccination compared to pre vaccination with a ratio <2 being considered as an insufficient response). The median vaccine-specific antibody response of the SAE patients (fold increase 3.97) did not differ significantly from that of the healthy controls (5.29; *P* = 0.90). The numbers were too small to investigate whether a difference exists between proximal and distal embolisation, but in two of the proximally embolised patients and none of the distally embolised patients, the ratio of the IgG antibody level post vaccination compared to pre vaccination was <2 [33•]. Foley et al. compared 38 patients who underwent proximal embolisation with 11 patients who underwent distal embolisation (and who were prospectively enrolled). Splenic function was measured in terms of IgM memory B cell numbers as a percentage of total B cells. There was no difference between the patients with proximal and distal embolisation (7.55 (6.11–9.31) vs. 9.97 (6.75–14.74)). However, there was a significant difference between the control group and the proximally embolised group (10.75 (9.58–12.06) vs. 7.55 (6.11–9.31)); *p* value 0.02) [34]. Currently, a multicenter trial is being conducted (by the Davis Medical Center of the University of California) in which a total number of 90 patients will be enrolled (30 patients treated with observation, 30 with embolisation, and 30 operatively treated patients) and compared with regard to splenic function, measured by geometric mean increases in pneumococcal antibody titre [35]. With the available evidence, we could say that routine vaccination is not indicated.

## Indications for Surgery

Whereas currently over 60–95% of the patients are treated non-operatively, accepted criteria for operative management have long been haemodynamic instability and associated intra-abdominal or pelvic injury requiring operative intervention. Time to intervention (to stop the bleeding) is another argument that is believed to be in favour of surgery. However, in our institution, we have compared time to embolisation with time to surgery. Median time to intervention did not differ, even in haemodynamically unstable patients (the median time to embolisation in haemodynamically stable patients was 177 min (IQR 78–233 min) vs 95 min (IQR 69–188 min) in the surgically treated patients and 46 min (IQR 27–107) vs. 64 min (IQR 45–80) in haemodynamically unstable patients) [36]. These data show that embolisation can safely be performed in haemodynamically unstable patients (defined as SBP ≤100 mmHg and heart rate >120 bpm on admission). It should be noted, however, that this concerns unstable patients with isolated blunt splenic injury. Also, an important requisite for this management is the 24-h availability of an interventional radiologist team and all the necessary resources. This should not be underestimated and remains to be the restrictive factor in wider implementation worldwide.

Different surgical techniques to control splenic bleeding exist, the classic (and most quick) method being splenectomy. Other techniques such as packing, splenorrhaphy, partial splenectomy, or the use of local haemostatic agents, such as Tachosyl^®^ or Floseal^®^, can also be used. In general, if the lesion is small or there is only a local bleeding site, haemostatic agents can be applied or the defect can be surgically sutured. For AAST grade IV or V injury, splenectomy is the surgical method of choice.

## Current and Future Perspectives

The trend towards NOM is evolving, and further refinement of patient selection is an important subject in current scientific research: who is a safe candidate for observation, who benefits from embolisation, and who needs (direct) surgical treatment. Although the presence of vascular injury is an indication for embolisation, new evidence is emerging that not all blushes require an intervention. Not only the location but also the size of a blush is important. Two research groups have published evidence that only blushes >1 and >1.5 cm correlate with the need for intervention [37, 38]. These findings should be confirmed in larger studies also including more diverse patient groups (i.e. also patients with blood clotting disorders or patients who take antithrombotic agents) before these criteria can be implemented in clinical practice.

**Fig. 1:**
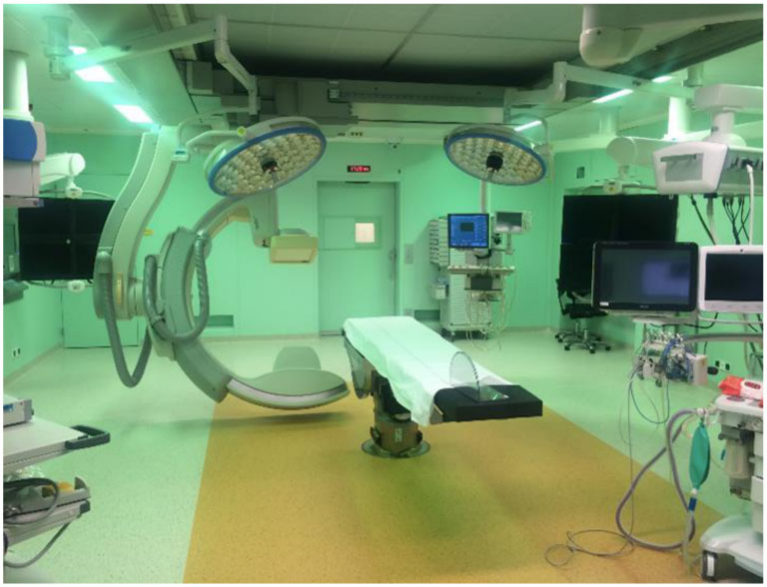
Example of a hybrid operating room in the Academic Medical Center in Amsterdam

We live in a world where rapid advancements are made, especially in technology. Nowadays, a number of hospitals have a hybrid operating theatre in place, where diagnostics and therapeutic (both endovascular and open surgical) techniques can be combined (see Fig. 1) [39]. This maximises patient’s benefit and allows us to stop the bleeding as quickly as possible according to one of the principles of trauma surgery: ‘Time is life’. Brenner et al. describe an interesting future perspective where the skill set of the acute care surgeon or trauma surgeon can be expanded with endovascular techniques. This results in surgeons themselves to perform splenic (or other abdominal/pelvic) embolisation [40], the big advantage being that the person performing the endovascular technique is also very familiar with polytraumatised patients, particularly when actively bleeding.

## Conclusion

The vast majority (60–90%) of the patients with splenic injury are treated non-operatively with success rates over 80%. A major advantage of NOM could be that splenic function is preserved as is suggested by the currently available literature (larger trials currently recruiting patients). However, disadvantages of NOM are the (small) risk of delayed splenic rupture, the possibility of a re-bleed, and the fact that no view is obtained of other abdominal organs. Especially in patients with multiple organ injuries, the latter should be considered.

Prognostic factors for failure of NOM include high-grade splenic injury grade (AAST grade 3 or higher), Injury Severity Score ≥25, and age ≥40 years. SAE can be used as an adjunct to observation in order to increase the success rate of NOM in patients with vascular injury (contrast extravasation, arteriovenous fistula, or pseudoaneurysm) or maybe even pre-emptively in patients with high-grade splenic injury (currently under investigation). Operative management is still indicated in case of haemodynamic instability (not responding to transfusion) or when associated intra-abdominal injuries requiring surgical treatment are present. It should be noted that in hospitals without sophisticated interventional radiological facilities (i.e. 24-h coverage of an experienced interventional radiologist and all the required resources), NOM should not be chosen over surgical management.

The challenge remains to stop the bleeding as quickly as possible by applying the most suitable or least invasive technique, reducing the number of unnecessary splenectomies in a safe manner (without increasing complication rate, readmission rate, or morbidity), thereby maximising patients’ benefit. Patient selection should be an ongoing subject of further research. Future studies should also focus on long-term outcomes (e.g. infectious episodes requiring antibiotic treatment or hospital admission, quality of life) of patients treated with embolisation.

## Appendix

**Table 1:** American Association for the Surgery of Trauma Organ Injury Scale Spleen (1994 revision)

Grade	Injury type	Description of injury
I	Haematoma	Subcapsular: <10% surface area
	Laceration	Capsular tear: <1 cm parenchymal depth
II	Haematoma	Subcapsular: 10–50% surface area; intraparenchymal <5 cm in diameter
	Laceration	1–3 cm parenchymal depth that does not involve a trabecular vessel
III	Haematoma	Subcapsular: >50% surface area or expanding; ruptured subcapsular or parenchymal haematoma; intraparenchymal haematoma >5 cm or expanding
	Laceration	>3 cm parenchymal depth or involving trabecular vessels
IV	Laceration	Laceration involving segmental or hilar vessels producing major devascularisation (>25% of the spleen)
V	Laceration	Completely shattered spleen
	Vascular	Hilar vascular injury with devascularised spleen
